# Spatial Multi-Omics in Alzheimer’s Disease: A Multi-Dimensional Approach to Understanding Pathology and Progression

**DOI:** 10.3390/cimb46050298

**Published:** 2024-05-20

**Authors:** Yixiao Ma, Wenting Shi, Yahong Dong, Yingjie Sun, Qiguan Jin

**Affiliations:** College of Physical Education, Yangzhou University, Yangzhou 225127, China; myixiao@163.com (Y.M.); wentingshi91@163.com (W.S.); dongyahong@139.com (Y.D.); sunyingj@outlook.com (Y.S.)

**Keywords:** spatial multi-omics, Alzheimer’s Disease, pathology and progression

## Abstract

Alzheimer’s Disease (AD) presents a complex neuropathological landscape characterized by hallmark amyloid plaques and neurofibrillary tangles, leading to progressive cognitive decline. Despite extensive research, the molecular intricacies contributing to AD pathogenesis are inadequately understood. While single-cell omics technology holds great promise for application in AD, particularly in deciphering the understanding of different cell types and analyzing rare cell types and transcriptomic expression changes, it is unable to provide spatial distribution information, which is crucial for understanding the pathological processes of AD. In contrast, spatial multi-omics research emerges as a promising and comprehensive approach to analyzing tissue cells, potentially better suited for addressing these issues in AD. This article focuses on the latest advancements in spatial multi-omics technology and compares various techniques. Additionally, we provide an overview of current spatial omics-based research results in AD. These technologies play a crucial role in facilitating new discoveries and advancing translational AD research in the future. Despite challenges such as balancing resolution, increasing throughput, and data analysis, the application of spatial multi-omics holds immense potential in revolutionizing our understanding of human disease processes and identifying new biomarkers and therapeutic targets, thereby potentially contributing to the advancement of AD research.

## 1. Introduction

Alzheimer’s Disease (AD) is characterized by its distinct pathological hallmarks in the brain, notably amyloid plaques and neurofibrillary tangles, which contribute to the progressive neurodegeneration observed in patients [1]. These pathological features are closely associated with the clinical manifestations of AD, including memory impairment, cognitive decline, and behavioral changes, making them central to the disease’s diagnosis and study [2]. At the molecular level, the amyloid cascade hypothesis has been pivotal in understanding AD’s pathogenesis, suggesting that the accumulation of beta-amyloid peptides initiates a series of events leading to synaptic dysfunction and neuronal death [3]. Despite significant advancements in elucidating AD’s molecular mechanisms, current treatments remain largely symptomatic, focusing on acetylcholinesterase inhibitors and NMDA receptor antagonists, with recent approvals of disease-modifying therapies sparking debate regarding their efficacy and accessibility [4]. The development of biomarker-based diagnostic methods, including cerebrospinal fluid analysis and amyloid PET imaging, has improved diagnostic accuracy, yet their application is hindered by high costs and invasive procedures [5]. The ongoing challenge in AD research and care underscores the need for a multifaceted approach that addresses the disease’s complexity and translates scientific insights into effective interventions [6].

The advancement of high-throughput “omics” sciences has fundamentally transformed our understanding of biological systems, unraveling the intricate mechanisms underlying disease pathogenesis and highlighting novel therapeutic targets [7]. Furthermore, the emergence of single-cell analysis techniques enables a more comprehensive understanding of the alterations in different cell types during the pathological process of AD, including neurons, astrocytes, microglia, and others, and their interactions during disease progression. Single-cell technologies can identify and analyze rare cell types and transcriptomic expression changes, providing novel insights into the investigation of early diagnosis and prognosis assessment of AD.

However, despite the significant potential of single-cell omics technology, there are challenges in addressing the spatial heterogeneity of AD. Firstly, AD is characterized by protein aggregation within specific brain regions, making it difficult for single-cell analysis to provide spatial distribution information about protein aggregation. Secondly, the pathogenesis of AD is influenced by intercellular interactions and the cellular environment, which single-cell analysis often fails to capture. In contrast, spatial omics solutions may be more suitable for addressing these issues in AD. Spatial omics techniques can provide spatial distribution information of cell types within tissues, allowing us to intuitively understand the relative positions and interactions of different cell types during the pathological process of AD.

Spatial multi-omics, integrating spatially resolved technologies such as spatial transcriptomics, proteomics, metabolomics and epigenomics, offers a comprehensive view of the molecular dynamics within tissues, elucidating the cellular heterogeneity and complex interactions within the microenvironment [8]. Building upon foundational omics technologies, recent advancements in spatial omics have notably enhanced the throughput and resolution. Some technologies now enable single-cell analysis, streamlining workflows to reduce operational times, and expanding the scope of analysis to encompass larger tissue areas and more diverse sample types. High-throughput methods now facilitate the analysis of thousands of genes (or whole transcriptome) across myriad single cells within a tissue section, providing a detailed cellular atlas of complex tissues in health and disease [9]. Innovations in imaging and sequencing technologies have driven the development of higher-resolution spatial omics, allowing for the dissection of cellular heterogeneity and the elucidation of microenvironmental influences at an unprecedented scale [10,11]. These advancements have significantly reduced the time required for data acquisition and analysis, enabling more efficient exploration of biological and pathological processes. Moreover, the expansion of the analysis area within spatial omics platforms has opened new avenues for comprehensive tissue mapping and the study of disease progression, particularly in complex disorders like Alzheimer’s Disease, where regional specificity is key to understanding pathology.

In conclusion, the advent of spatial omics technologies marks a pivotal advancement in biomedical research, providing unparalleled insights into the molecular composition of biological tissues with precise spatial resolution. This review encompasses the technological developments in proteomics, transcriptomics, epigenomics, and metabolomics in spatial omics, while also summarizing some applications of spatial omics in Alzheimer’s Disease research, paving new pathways for research into AD. Spatial omics is instrumental in mapping the spatial dynamics among biomarkers, cellular identities, and pathological features in AD, thereby facilitating early diagnosis and the advent of tailored therapeutic approaches. This review will delve into emerging spatial omics technologies and their application in AD research (see Table 1).

## 2. Spatial Omics Methods

### 2.1. Space Proteomics Techniques

Protein imaging is indispensable in understanding cell functions, disease research, therapeutic target identification, and other areas of scientific inquiry. Techniques such as Immunofluorescence, Live-cell Imaging, Western Blotting, Gel Electrophoresis, and X-ray Crystallography, along with advanced methods like Mass Spectrometry, Immunohistochemistry (IHC) and Proximity Ligation Assay (PLA), offer diverse insights into protein structure, function, and location. Each method has its strengths and can be used alone or synergistically in biological research. Despite their utility, many traditional methods have limitations in balancing high-throughput techniques with in situ imaging or identifying a broad range of proteins. Spatial proteomics technologies address these challenges. They are generally suitable for both fresh-frozen and formalin-fixed paraffin-embedded (FFPE) samples and fall into two main categories, cyclic fluorescent approaches and Mass Spectrometry Imaging (MSI). Cyclic fluorescent techniques, while offering high resolution at the single-cell or subcellular level, have limited multiplexing capabilities (typically around 50~100 proteins). In contrast, MSI can process a wide variety of sample types and generate extensive data sets. It is capable of detecting multiple molecules simultaneously, including proteins, lipids, drugs, and their metabolites. However, the complexity of data processing and the spatial resolution limits, dictated by the instrumentation’s capabilities, are significant considerations in MSI [32].

#### 2.1.1. Cyclic Fluorescent Imaging

The cyclic immunofluorescence approach was first described by Gerdes et al. [33]. Cyclic fluorescent methodologies facilitate protein visualization through antibody binding, using either fluorophores or DNA barcodes for the tagging process. These highly multiplexed imaging techniques adopt diverse detection strategies to circumvent the spectral constraints inherent in traditional fluorescence microscopy. This advancement significantly enhances our capability to concurrently analyze up to 100 proteins in both FFPE and fresh-frozen tissue samples. One of the strategies of these fluorescent cyclic approaches involves a repetitive process of staining, imaging, and then either bleaching or antibody removal, such as MP-IHC and t-CycIF. MP-IHC is a method for multiplexed immunohistochemistry that uses commercially available reagents and conventional epifluorescence. It increases throughput by labeling up to ten antibodies consecutively every cycle, with no restriction on the number of cycles [19]. CycIF is likewise based on a technology for visualizing proteins in cells or tissues using highly multiplexed immunofluorescence imaging. A fluorescent substance attaches to a certain protein, the fluorescence signal is deactivated, and then another molecule binds to a different protein in a cyclical process. This procedure is carried out numerous times to find various proteins, and the photos are then processed to show the cellular architecture. t-CycIF employs readily accessible chemicals, standard slide scanners and microscopes, manual or automated slide processing, and straightforward techniques. As a result of its adaptability, cyclic fluorescent methodologies can be utilized in most research or clinical laboratories using existing equipment, offering a significant advantage in terms of accessibility and practicality [12]. Nevertheless, these methodologies, irrespective of their manual or automated application, are impeded by critical limitations. These include the verification of antibody sample loss and epitope destruction.

Another group of fluorescent cyclic approaches relies on DNA barcodes. These methods utilize single-stranded locked nucleic acids (ssLNAs), single-stranded DNA (ssDNA), or DNA-based antibodies or probes. This approach significantly accelerates the process, as all antibodies can be bound in a single step. Immuno-SABER uses DNA-barcoded antibodies and orthogonal DNA concatemers produced by primer exchange reactions (PERs) to accomplish signal amplification. It provides customizable signal amplification without enzymatic processes and is scalable for concurrently amplifying and viewing many targets [14]. In PRISM using ssDNA-conjugated antibodies or peptides, markers are barcoded with single-stranded nucleic acid oligonucleotides (docking strands), which are rationally designed to optimize orthogonality between complementary fluorescently labeled ssLNA or DNA imaging probes used for confocal or super-resolution imaging [34]. Following marker and imaging probe validation, multiplexed imaging is performed using sequential labeling and washing out of individual imaging probes (or dehybridization of fluorophore), with wash-steps in between used to clear the sample of imaging probes or fluorophore removal. These signal amplification methods enhance throughput by allowing simultaneous application of multiple primary antibodies, reducing both sample preparation and image acquisition times.

#### 2.1.2. Mass Spectrometry Imaging

Imaging Mass Spectrometry (IMS, also known as Mass Spectrometry Imaging (MSI)) is a sophisticated analytical method that combines Mass Spectrometry’s power with spatial information. In an IMS experiment, a probe—which might be a laser, an ion beam, or a liquid junction—is rastered over a surface to desorb or extract biomolecules, such as proteins, lipids, metabolites, and medications—which are then immediately evaluated by Mass Spectrometry (MS). IMS allows for the examination of a sample using Mass Spectrometry while maintaining its spatial context. This is accomplished by building a two-dimensional molecular map by methodically collecting mass spectra at discrete positions over the sample’s surface. IMS’s adaptability encompasses a variety of strategies, including Matrix-Assisted Laser Desorption/Ionization (MALDI), Secondary Ion Mass Spectrometry (SIMS), and Desorption Electrospray Ionization (DESI), among others. MALDI-IMS has gained popularity due to its compatibility with a diverse variety of analytes and sample types, including biological tissues, thin slices, and whole-body samples [35].

IMS’s adaptability is highlighted through various strategies such as MALDI, SIMS, and DESI. MALDI-IMS is particularly popular due to its compatibility with a wide range of analytes and sample types, including biological tissues and whole-body samples. The technique facilitates in situ molecular mapping without specialized reagents, using a chemical matrix to enhance sensitivity for desired analytes. This matrix, crucial for absorbing photons from the MALDI laser, is applied to thinly sectioned tissue samples, enabling rasterized mass spectra production for precise spatial analysis, achieving resolutions down to 20 μm [35].

DESI, introduced in 2004 [36], focuses on enhancing the ionization process’s sprayer mechanism. These advancements, such as the strategic positioning of the solvent capillary within the gas capillary or nozzle, have improved control and reproducibility. This evolution in DESI technology has facilitated high-resolution imaging on tissue samples and compatibility with fast-scanning mass spectrometers, thereby streamlining data collection and bolstering its utility in swift and reliable clinical diagnoses [13]. Building upon these developments, Nano-DESI emerges as an ambient pressure ionization technique that employs minimal solvent for precise desorption and ionization. Its high sensitivity and the elimination of sample preparation requirements enable ambient imaging with resolutions surpassing 12 μm [37]. Further, the integration of Nano-DESI with Ion Mobility (IM) separation represents a progressive step, enhancing the spatial analysis of intricate biological samples by removing interferences and ensuring accurate measurements, thus increasing molecular specificity [20]. Multiplexed Ion Beam Imaging (MIBI) utilizes Secondary Ion Mass Spectrometry to visualize metal-isotope labeled antibodies, enabling simultaneous analysis of samples stained with up to 100 antibodies. Compatible with formalin-fixed, paraffin-embedded tissue sections, MIBI offers sensitivity and resolution on par with high-magnification light microscopy. Its application in tissue analysis facilitates detailed classification and insights, underscoring its utility in clinical research [38,39]. Post-MIBI’s introduction, the development of multiplexed ion beam imaging by time of flight (MIBI-TOF) technology has notably enhanced channel multiplexing and reduced data acquisition times. This technology enables automated imaging with resolutions up to 260 nm, covering extensive fields for in-depth tissue analysis [40].

In the realm of spatial omics, the application of sample surface treatment techniques before Mass Spectrometry analysis emerges as an effective strategy for the effective selection of target regions. Among these, Laser Capture Microdissection (LCM) stands out as a method for procuring specific subpopulations of tissue cells under microscopic visualization. This technology enables the selective isolation of particular cells or tissues from heterogeneous samples, which is particularly valuable in fields like pathology, where analyzing specific cell populations within a diverse tissue matrix is crucial. The cells separated by LCM can subsequently be subjected to a range of molecular analyses, including DNA, RNA, or protein assays. The integration of LCM with Mass Spectrometry, forming LCM-MS, enhances both the accuracy and spatial resolution of these analyses [15].

Concurrently, NanoPOTS and microLESA emerge as innovative technologies that complement these processes. NanoPOTS, with its robotic nanopipetting, microfabricated glass nanowell chips, and efficient one-pot processing, enables sample preparation in volumes as small as 200 nL. This minimizes sample loss due to adsorption and maximizes protein concentration for digestion. In combination with nanoLC-MS/MS, NanoPOTS facilitates the identification of nearly 700 proteins from individual cells [41]. Furthermore, when applied alongside LCM, NanoPOTS allows for targeted protein profiling from specific tissue regions, even from a limited number of cells (as few as 10). However, its wider application in high-resolution proteome imaging is currently restrained by manual processing and the lack of sophisticated analysis tools [21,42]. microLESA has made significant strides in proteome profiling. This method involves depositing minuscule enzyme droplets, approximately 250 pL in size, onto tissue surfaces for tryptic digestion. Critical experimental parameters, including tissue thickness, trypsin concentration, and enzyme incubation time, have been meticulously evaluated and optimized to enhance the efficacy of proteomics analysis [18].

### 2.2. Spatial Transcriptomic Techniques

Spatial transcriptomic tools can be categorized into two main types, sequencing-based and in situ imaging-based modalities. Sequencing methods involve tagging transcripts with an oligonucleotide address that indicates their spatial location. This is most commonly achieved by placing tissue slices on a barcoded substrate, such as Visium and Stereo-seq, among others. On the other hand, in situ imaging methods typically employ variations of fluorescence in situ hybridization (FISH), examples of which include Xenium and MERFISH. Ideally, spatial transcriptome data should offer genome-wide expression measurements that are both spatially resolved and precise at the single-cell level. However, due to technical constraints, most spatial transcriptomics assays must compromise either spatial resolution or gene coverage. For instance, in situ capture and sequencing-based techniques can capture any mRNA molecules without prior knowledge of their sequences, but they generally do not provide single-cell level spatial resolution [9]. In contrast, in situ sequencing and FISH-based mRNA measurement methods can achieve cellular or subcellular resolution. Yet, these assays typically have a limited throughput, detecting only a specific range of genes (usually between 30 to 500) and necessitate prior knowledge for the design of probes [11,43,44].

In the field of spatial transcriptomics, a subset of technology, the initial step involves positioning numerous primers, each marked with a distinct barcode, on a microscopic glass slide, thereby forming a microarray. These spots, identifiable by their unique barcodes and precise locations, enable the determination of the original tissue location of each RNA sequence. Subsequently, a tissue section is placed atop this microarray. This setup facilitates the interaction of transcripts from the tissue with the fixed cDNA synthesis primers during a reverse transcription (RT) reaction. The synthesized cDNA library is then sequenced, and the obtained data are correlated with the spatially organized spots. Following this, the data are integrated with a high-resolution histological image of the tissue section. The detail of the resolution is contingent upon the spot’s dimensions. For instance, Slide-seq employs DNA-barcoded beads measuring 10 μm [26], whereas Visium features a spot size of 55 μm. Although achieving sub-histological resolution is feasible, the potential loss of information between spots might overlook critical data from rare cells [45].

However, Stereo-seq, which combines DNA nanoball (DNB)-patterned arrays with in situ RNA capture, overcomes these constraints. The standard DNB chips in Stereo-seq feature spots about 220 nm in diameter, with a center-to-center distance of either 500 or 715 nm, thus providing up to 400 spots for tissue RNA capture per 100 μm^2^. This technique offers a significant advancement by enabling single-cell resolution, increased sensitivity, and a broader field of view, covering an effective area of 13.2 cm × 13.2 cm [27].

Additionally, there are several techniques that utilize microfluidic deterministic barcoding strategies, including DBiT-seq [22], spatial-ATAC-seq [46], and spatial-Cut & Tag [47]. DBiT-seq, which stands for Deterministic Barcoding in Tissue for spatial omics sequencing, is particularly notable for its capacity to co-map mRNAs and proteins on a formaldehyde-fixed tissue slide using Next-Generation Sequencing (NGS). This method employs parallel microfluidic channels to transfer DNA barcodes onto a tissue slide. The crossflow of two barcode sets, A1~50 and B1~50, followed by in situ ligation, results in a two-dimensional mosaic of tissue pixels, each with a unique composite AB barcode. This innovative approach was applied to mouse embryos, unveiling significant tissue types in early organogenesis, as well as detailed structures such as the microvasculature in the brain and pigmented epithelium in the eye field. Remarkably, the gene expression profiles captured in 10 μm pixels align with clusters of single-cell transcriptomes, enabling swift identification of cell types and their spatial distributions [22].

FISH tags mRNA molecules with hybridization probes. These probes are detected combinatorically over multiple rounds, involving staining with fluorescent reporters, imaging, and de-staining. This technique embodies the high spatial resolution and sensitivity of FISH, providing single-cell resolution data. Different spatial transcriptomics platforms utilize varied protocols, probe designs, signal amplification strategies, and computational processing methods, potentially leading to differences in sensitivity and downstream results [48].

After the preparation of tissue sections, Xenium’s padlock probes, which consist of two arms, must both stably hybridize to their target for stability. If only one arm hybridizes, the probe becomes unstable and is washed off during the post-hybridization process. These probes also contain a gene-specific barcode sequence, generating a unique optical signature for each transcript. Stable hybridization of both probe arms with a perfect match initiates proximal probe ligation, followed by rolling circle amplification. The amplified products undergo successive rounds of fluorescent probe hybridization, imaging, and removal, creating an optical signature specific to each gene. This process allows for the construction of a spatial transcript map across the entire tissue section [49].

The design of Spatial Molecular Imager (SMI) probes involves two key components, in situ hybridization (ISH) probes and fluorescent reporters. ISH probes, consisting of a target-binding domain (35~50 nt DNA) and a readout domain (60~80 nt DNA), hybridize with specific RNA targets in tissue samples. Each readout domain accommodates four unique fluorescent reporters, enabling the detection of multiple RNA sequences. In the assay, FFPE tissue samples undergo cyclic readouts using sets of these fluorescent reporters in an SMI instrument. This process captures high-resolution Z-stacked images for each cycle, where fluorophores are sequentially hybridized, imaged, and then removed before the next cycle. The analysis workflow includes 3D image processing to identify reporter spots, decoding of RNA transcripts, cell segmentation using DAPI and antibodies, and assigning RNA transcripts to individual cells [50].

MERFISH, a highly multiplexed in situ hybridization imaging method, significantly increases the capacity for simultaneous imaging of multiple RNA species in single cells. It achieves this through combinatorial labeling and sequential imaging with error-robust encoding schemes. RNA transcripts are targeted and labeled using a preselected gene panel, assigning each transcript a unique binary barcode that is error-robust, thus ensuring reliable transcript identification, even among hundreds of genes. The method involves hybridizing a sample with encoding probes that imprint barcodes onto each RNA species. These barcodes are then detected through sequential rounds of multichannel imaging, using subsets of fluorescently labeled readout probes that hybridize with the barcode region of the encoding probes. Fluorescent spots are computationally decoded into binary barcodes, with the presence of a spot indicating 1″ and its absence 0″. These barcodes, along with their intracellular positions, are combined with cell nucleus and boundary staining, enabling single-cell resolution gene expression measurement [11].

Some spatial omics techniques involve imaging the tissue to be tested, followed by cutting out the region of interest (ROI), such as with LCM [25] and UV photolysis [24] technology. These methods are then combined with sequencing technologies to yield the corresponding omics data. This approach is akin to more targeted sequencing of bulk groups.

Digital Spatial Profiling (DSP) is a highly multiplexed method for spatial profiling of proteins or RNAs, suitable for FFPE samples. This approach utilizes affinity reagents such as antibodies or RNA probes, which are covalently linked to a DNA indexing oligonucleotide through a UV-cleavable linker (PC-oligo). These reagents are employed to stain tissue sections, and focused UV light is used to release the indexing oligonucleotides from any ROI. Subsequently, these oligonucleotides are collected and digitally counted or quantified via NGS. This technique enables multiplex digital spatial profiling of proteins and RNA in fixed tissue. While the official recommendations suggest a range covering 1 to ~5000 cells, practical applications typically favor a middle range, and it does not achieve single-cell level analysis [24].

SMI represents a system designed for the measurement of RNAs and proteins within intact biological samples, achieving subcellular resolution through multiple cycles of nucleic acid hybridization employing fluorescent molecular barcodes. The chemistry of SMI is reliant on the integration of in situ hybridization (ISH) probes, or alternatively antibodies, paired with fluorescent readout probes. SMI has demonstrated detection of up to 980-plex RNA and up to 108-plex proteins. For RNA profiling, these 980 panels facilitate the elucidation of pivotal cellular states, encompassing immune cell states, apoptosis, autophagy, stress responses, and damage responses, as well as delineating intricate cell–cell interactions and hormone activity [50].

#### Overview of Post-Experimental Analysis in Spatial Transcriptomics

The rapidly increasing volume of spatially resolved transcriptomics (SRT) data poses challenges in terms of inconsistent downloading protocols and processing steps. Traditionally, accessing these data from academic publications can be cumbersome due to unfriendly user interfaces, large raw sequencing data files, time-consuming processing steps, and frequent issues with data link maintenance, which may hinder access and compromise reproducibility. To address these issues and improve data accessibility for bioinformatics researchers, several databases and web servers have been developed, including cellxgene (https://cellxgene.cziscience.com/, accessed on 3 May 2024), SpatialDB [51], STOmicsDB [52], SODB [53], and SOAR [54].

Additionally, spatial transcriptomics requires innovative approaches for data analysis. Computational workflows vary depending on the technology used to produce the data, but there is a significant overlap in how different modalities are processed. The goal of these workflows is to correlate various signals—whether from fluorescent intensities, barcodes, the sequences of reverse-transcribed RNA, or the *m*/*z* values of ions—with specific spatial locations within the tissue. Technologies that achieve cellular resolution can assign these signals to individual cells through a process known as cell segmentation.

Cell segmentation involves delineating cell boundaries and other structures such as the nucleus. Although this task might seem straightforward to the human eye, automating it has proven challenging, especially in environments like solid tumors or with cells displaying complex shapes like neurons. Deep-learning-based segmentation algorithms, such as Cellpose 2.0, Mesmer, and Segment Anything, have outperformed traditional methods like watershed, although they remain highly sensitive to variations in cell diameter and shape.

In the context of AD, the neuronal environment is intricate and dynamic, where identifying molecules and understanding cellular communications could deepen our neurobiological understanding of AD and potentially reveal new therapeutic opportunities. Similarly, spatial transcriptomics (ST) analysis aims to connect and integrate gene expression data with specific cellular or transcript locations. This technology enables the precise identification and localization of cell types. Commercial ST technologies like 10x Genomics Visium and NanoString GeoMX, which typically lack single-cell resolution, use methods such as enrichment analysis [55] and deconvolution [56] to estimate cell-type compositions, enhancing the quantitative assessment of cellular distribution. Techniques such as multiplexed FISH combined with unsupervised clustering and annotation, similar to those used in scRNA-seq, allow for unbiased cell type identification, even at single-cell resolution [10,57]. Furthermore, newer computational approaches have emerged to reconstruct spatial information from scRNA-seq data, optimizing spatial correlations and providing both probabilistic and deterministic alignments for enhanced mapping and analysis [58,59].

The pathogenesis of AD extends beyond neurons alone. Increasing evidence suggests that interactions among multiple cell types within the brain play a crucial role in promoting the development of AD [60]. A key objective of ST analysis is to investigate how cells interact with their surrounding tissue environment. In spatial transcriptomics, innovative computational methods are employed to analyze cell–cell interactions and gene expression. Techniques such as the two-way comparison by Giotto [55] and similar approaches in CellPhone DB v3.0 [61] leverage spatial information to reduce false positives in ligand–receptor predictions. Cell2location [62] is used to handle data without single-cell resolution by inferring cell-type locations for gene expression comparison. Other methods include convolutional neural networks, optimal transport, and multioutput regression to assess the impact of neighboring cell types. Furthermore, advanced algorithms like NCEM [63], CLARIFY [64], SpatialDM [65], and COMMOT [66] integrate receptor–ligand analysis with spatial data to deepen our understanding of cellular processes, although their comparative effectiveness remains to be evaluated.

### 2.3. Spatial Epigenomic Techniques

Spatiotemporal control of gene expression, pivotal for cell and tissue development and function, is intricately governed by the regulatory information encoded in the epigenome. This regulation, including vital histone and DNA modifications, facilitates differential gene activation or repression, leading to the formation of diverse cell types during development [67]. Traditional sequencing-based approaches, now extended to single-cell level, have made strides in profiling these epigenetic modifications and chromatin accessibility [68]. However, these methods, requiring cell dissociation, often overlook the spatial context crucial for understanding epigenomic influence in tissue-specific development. For instance, during embryonic brain development, spatial patterns of morphogenic gradients and transcription factors crucially guide the differentiation of neural progenitors [69,70].

Epigenomic MERFISH addresses this gap by capturing specific epigenetic modifications on chromatin in situ. It involves tagging DNA with T7 promoters near these modification sites, followed by in situ transcription to generate RNAs, and finally, detection of these RNAs via MERFISH. This technique allows for transcriptomic-scale RNA imaging and high-resolution profiling of epigenetic modifications, including those marking active promoters, enhancers, and silent chromatin regions in individual cells. Importantly, it can image histone modifications on genomic loci as short as a few hundred bases, offering a resolution of less than 1 kb. This high spatial resolution enables researchers to determine sub-nuclear localizations of genomic loci and their spatial relationship with nuclear structures, a critical aspect of spatially resolved epigenomic profiling in complex tissues [31].

In addition, ATAC–RNA-seq provides a diverse perspective. This technique starts with fixing a frozen tissue section with formaldehyde, followed by treatment with a Tn5 transposase complex preloaded with a DNA adaptor for integration into accessible genomic DNA sites. After incubation with a biotinylated DNA adaptor binding to mRNA, spatial barcodes are introduced via a microfluidic chip, creating a two-dimensional grid of uniquely barcoded tissue pixels. Following reverse crosslinking, barcoded DNA, including cDNA and gDNA, is extracted and sequenced. Spatial CUT&Tag–RNA-seq, a variant of this method, involves targeting specific histone modifications with antibodies and a protein A-tethered Tn5-DNA complex for CUT&Tag, mirroring the steps of ATAC–RNA-seq and culminating in the spatial co-profiling of histone modification occupancy and the transcriptome [30]. However, the spatial epigenome has not been reported in revealing the pathology and progression of AD.

## 3. Applications of Spatial Omics in AD

Spatial omics presents unique advantages over techniques such as proteomics, single-cell sequencing, and bulk sequencing. Through spatial omics, we can concurrently acquire spatial information regarding protein/gene/epigenetic expression at both tissue and cellular levels, elucidating interactions between cells and tissue structures. Consequently, this provides a more comprehensive understanding of tissue functionality and complexity. Furthermore, spatial omics not only facilitates the identification of cell types and subtypes but also enables the examination of spatial distribution among cells, interactions among neighboring cells, and cellular positioning within tissues, thereby offering invaluable insights for in-depth exploration of biological processes. In comparison, while single-cell sequencing furnishes gene expression data for individual cells, it lacks spatial relationships between them. Similarly, bulk sequencing furnishes average expression data for entire tissues but fails to discern differences between cell types and regions. The following section encapsulates the pivotal findings from recent studies, accentuating the contributions of various spatial omics technologies towards advancing the understanding of AD. Furthermore, a summary of spatial omics technology applications in AD is presented in Table 2.

### 3.1. Application of Spatial Proteomics in the Study of AD

Spatial proteomics is crucial for elucidating the pathology of AD. Amyloid β (Aβ) deposition, an early and invariable feature of AD, consists of peptides generated from amyloid precursor proteins (APPs) by β- and γ-secretases. Despite its significance, the distribution of individual Aβ peptides in the brains of aged individuals, those with AD, and cerebral amyloid angiopathy (CAA) is not fully characterized. To address this gap, Matrix-Assisted Laser Desorption/Ionization–Imaging Mass Spectrometry (MALDI-IMS) has been employed, revealing that Aβ1–42 and Aβ1–43 are selectively deposited in senile plaques, while full-length Aβ peptides such as Aβ1–36 to Aβ1–41 are found in leptomeningeal blood vessels. Moreover, this approach has enabled the visualization of distinct depositions of N-terminal truncated Aβ40 and Aβ42 species, underscoring the significance of even minor variations in peptide structure [81]. In parallel, the investigation of synaptic proteins provides further insights. Through 13-channel imaging of cultured rat hippocampal neurons using ssLNA imaging probes and non-PRISM fluorescent markers, the complex network of synaptic and cytoskeletal proteins central to synapse formation and plasticity has been characterized. This includes key proteins like actin, Tuj-1, MAP2, synapsin-I, and VGLUT1, illustrating the diverse aspects of brain development and neuronal circuit function [34]. Additionally, the precise delineation of the anterior olfactory nucleus (AON) in neurodegenerative studies is critical due to its specific accumulation of pathological aggregates such as α-synuclein, tau, β-amyloid, huntingtin, and TAR DNA-binding protein 43 (TDP43) [102,103,104,105,106]. MP-IHC using a pixel-bin approach facilitates the assessment of different tissue structures, including blood vessels, white matter, and olfactory bulb layers. This technique, which utilizes markers not confined to nuclear or cytoplasmic distributions, is instrumental in identifying unique neurochemical signatures for each bulb layer, crucial for the reliable and consistent delineation needed in neurodegenerative research [107].

### 3.2. Application of Spatial Metabolomics in the Study of AD

Spatial metabolomics in AD offers a nuanced understanding of brain lipid dynamics, crucial for cellular signaling and neuronal function [108]. Studies have shown a decrease in various fatty acids such as LA, OA, EPA, AA, DHA, and DPA in the brains of humans and AD animal models. This change in metabolism of free fatty acids (FFAs) is significant in understanding the most common form of dementia [109,110]. Advanced techniques like DESI and MALDI-MS imaging have detected significant changes in lipid metabolites, such as phosphatidylethanolamines, phosphatidylcholines, and glycerophospholipids, in early-stage AD mouse models [111]. These techniques have also been instrumental in observing the distribution of specific lipids like DHA in dementia mouse models [75] and studying the enzymatic activity of biomarkers like BChE in AD patients [112]. Lipid molecules, including lysophospholipids such as LPC and LPG, accumulate in Aβ plaques, affecting cell membrane functions like ion transportation [113] and signal reception [113]. Metabolites like AA [114], critical for synaptic signaling, are also found in Aβ plaques. The altered expression of various metabolites, including lysophospholipids, spermine, and malic acid, is closely linked to AD development [115]. The role of lipid molecules in cell membrane composition and neurotransmitter signaling in neurodegenerative diseases like AD is now evident [116]. Lipids are involved in amyloidogenesis by regulating APP, BACE1, and γ-secretase complex components [117]. Sulfatide (ST) is a sphingolipid with an important role in the central nervous system as a major component of the myelin sheath. Techniques like MALDI-IMS have been used to assess differences in lipid species in different stages of AD, revealing depletion of sulfatide lipid species from early stages of the disease [16]. Furthermore, transgenic Alzheimer’s Disease mice models have shown brain region-specific differences in Aβ peptide aggregation, with significant accumulation of Aβ species in plaques, highlighting the complex interplay of lipids in AD pathology [118].

### 3.3. Application of Spatial Transcriptomics in the Study of AD

Spatial transcriptomics is enhancing our understanding of AD by revealing transcriptional alterations in the brain’s affected regions. Chen and colleagues illustrate the application of spatial transcriptomics [9] and in situ sequencing [119] technologies. In an AD mouse model, this study investigates the transcriptional changes in tissue domains within a 100 μm diameter around amyloid plaques using spatial transcriptomics. Early alterations were observed in a gene co-expression network enriched with myelin and oligodendrocyte genes (OLIGs)In the later phase of the disease, a multicellular gene co-expression network involving 57 plaque-induced genes (PIGs) — related to the complement system, oxidative stress, lysosomes, and inflammation — became prominent.. Similar alterations in human brain samples partially strengthen these observations, suggesting that genome-wide spatial transcriptomics analysis can untangle the dysregulated cellular network near AD’s pathogenic hallmarks and other brain diseases [120]. Another study utilized the 10× Genomics Visium platform to enhance the resolution to 55 μm. In conjunction with co-immunofluorescence staining for AD-associated pathological markers, this approach enabled a detailed delineation of gene expression within the human middle temporal gyrus (MTG), known for its early vulnerability in AD. By annotating cortical layers and white matter (WM) in both AD and control MTG samples, the team identified specific marker genes for five cortical layers and the WM. They discovered layer-specific differentially expressed genes (DEGs) in human AD patients compared to the controls, including previously identified markers like RAR-related orphan receptor B (RORB), Purkinje cell protein 4 (PCP4), Myelin basic protein (MBP), and novel marker genes such as secreted protein acidic and rich in cysteine (SPARC), Calbindin 2 (CALB2), DIRAS Family GTPase 2 (DIRAS2) and Keratin 17 (KRT17) [28]. Moreover, the early signs of AD, such as poor short-term memory and olfactory dysfunction, were explored through spatial transcriptomics in the hippocampal and olfactory bulb (OFB) regions of 3 × AD, 3 × PB, and control mice. The study found global deregulation in genes like Ubc, Pkm, Cox6c, and Glo1, affecting cellular metabolism and energy production. Notably, the upregulation of Gabra2 in the hippocampus and Gabra5 in the OFBs in AD strains highlighted their role in GABAergic synaptic balance, with Bok identified as a differentially expressed gene in both mouse and human AD brains, implicating mitochondrial function and apoptosis. This research emphasizes the significance of these genes in AD pathology and their potential as biomarkers for early AD detection [121].

## 4. Conclusions and Challenges

Over the past decade, the rapid advancements in NGS technologies have propelled significant progress in the field of multi-omics, extending some omics analyses down to the single-cell level. However, sequencing-based methods that require cell dissociation can lead to a loss of crucial spatial information, as the original spatial context of cells is compromised, which is particularly vital in omics studies. This loss is especially pertinent in AD research, where cell-type-specific changes, including neurons, astrocytes, microglia, and their spatial distribution and interaction patterns within the brain, are crucial for understanding disease development. The characteristic pathological features of AD, such as Aβ plaques and tau neurofibrillary tangles, exhibit distinct spatial distribution patterns across different brain regions. These features are associated with damage to specific neural pathways that span multiple areas of the brain. Additionally, the progression of AD is closely linked to changes in the brain’s microenvironment, including inflammatory responses, compromised integrity of the blood–brain barrier, alterations in intercellular communication, and changes in metabolic pathways and biochemical processes that may be confined to specific regions or cell groups within the brain.

This article provides an overview of the current spatial omics technologies and their advancements, as well as their existing applications in AD research, highlighting the significance of spatial omics in detecting AD pathological features among other aspects. Despite significant advancements, several challenges and issues remain.

Spatial multi-omics encounters several challenges. Firstly, standardized sample preparation is crucial for successful experiments and high data quality. Tissue handling requires adherence to standardized protocols for tissue sampling, fixation, freezing, and tissue sectioning. Particularly, brain tissue exhibits intricate anatomical structures and diverse cell types, necessitating more cautious handling compared to other tissue types due to its softness and vulnerability.

For antibody-based multiplex imaging methods, the specificity of antibody panels is associated with increased costs. In cases requiring custom antibodies or antibody combinations, their validation may be time-consuming, and antibody specificity may often be ambiguous. Moreover, this imaging method depends on the number and coding of fluorescent channels, making it crucial to enhance throughput and shorten formal experimentation time. Additionally, techniques for sample surface treatment and selection of regions of interest (ROIs), such as sampling of amyloid plaques and surrounding areas separately, can provide spatial positioning at a lower precision. However, in sequencing analysis, this is akin to a small bulk sample, making it difficult to achieve single-cell resolution. Furthermore, certain high-throughput spatial techniques do not achieve single-cell resolution, and single-cell assays, despite their precision, are limited by throughput and the need for fixed detection panels, making custom probe design prohibitively expensive (refer to Table 3 for a comparative analysis of other technologies in spatial omics).

Lastly, for subsequent analysis of spatial omics data, the sheer volume of generated data is immense, requiring robust data storage strategies that may be inaccessible to many academic laboratories. Moreover, in the subsequent analysis phase, most researchers rely on specialized bioinformatics departments for collaborative research. These issues necessitate more user-friendly and convenient data storage and analysis methods.

## 5. Future Direction

Spatial omics techniques offer both opportunities and challenges for a deeper understanding of Alzheimer’s Disease (AD) pathology and progression. Firstly, with the continuous development and refinement of technology, we anticipate the emergence of higher-resolution, faster, and more accurate imaging techniques, such as spatial single-cell resolution multi-omics technologies. This advancement will enable a more detailed investigation of the spatiotemporal characteristics of AD-related neuronal damage, protein aggregation, and pathological changes. Anticipated progress includes increased throughput, reduced costs, integration of more modalities per detection, and improved sensitivity and specificity.

Furthermore, emerging artificial intelligence (AI) methods hold the potential to propose new avenues for the application of spatial omics in understanding the therapeutic effects and mechanisms of action of AD treatments. AI models include machine learning (ML)-based [122], deep learning-based, and network-based algorithms [123]. Compared to traditional biological experiments, AI-based models have demonstrated faster and more effective outcomes by leveraging large-scale biomedical data. Specifically, through spatially tracking drug distribution and effects in brain tissue, we can more effectively assess the efficacy and side effects of drugs and provide more reliable experimental data for drug development. On one hand, this can be achieved through AI-driven drug screening, prediction of drug mechanisms of action, and drug repurposing technologies. On the other hand, through the development of AI algorithms for enhancing the analysis and interpretation of spatial omics data. Therefore, AI-based computational methods can provide robust support from multiple perspectives for AD drug target research and drug development.

## Figures and Tables

**Table 1:** Comparative overview of spatial omics technology.

Platform	Spatial Resolution	Molecular Class	Throughput	Image Area	Molecular Identity Confirmation Method	References
CyCIF	1 µm	Protein	<100	Slide Area	Fluorescence Imaging	[12]
DESI IMS	50 µm	Metabolite, Lipid, Protein	/	50 μm × 50 μm	MS	[13]
Immuno-SABER	100 μm	Protein	<50	1 mm × 1 mm	Fluorescence Imaging	[14]
LCM-MS	10–50 μm	Metabolite, Lipid, Protein	<250	Slide Area	MS	[15]
MALDI IMS	15 μm	Metabolite, Lipid, Protein	/	Slide Area	MS	[16]
MIBI-TOF multiplex ion-beam	260 nm	Metabolite, Lipid, Protein	<50	Slide Area	MS	[17]
microLESA	110 μm	Protein	>2000	Slide Area	MS	[18]
MP-IHC	10 µm	Protein	>100 Proteins	10 μm × 10 μm	Fluorescence Imaging	[19]
Nano-DESI MSI	10 µm	Metabolite, Lipid, Protein	/	Slide Area	MS	[20]
NanoPOTS	100 µm	Protein	>2000 Proteins	Slide Area	MS	[21]
DBiT-seq	10 μm	RNA, Protein	<50 Proteins, Whole Transcriptome	1 mm × 1 mm	Sequencing	[22]
CosMX SMI	50 nm	RNA, Protein	<200 Proteins, <1000 RNA	range 16–375 mm^2^	Fluorescence Imaging	[23]
GeoMX DSP	50 μm	RNA, Protein	<2000	35.3 mm × 14.1 mm	Sequencing	[24]
LCM-seq	10–50 μm	RNA	<15000	Slide Area	Sequencing	[25]
MERFISH	0.5 μm	RNA	<1000	40 μm × 40 μm	Fluorescence Imaging	[11]
Slide-seq	10 μm	RNA	Whole Transcriptome	Slide Area	Sequencing	[26]
Stereo-seq	0.5 μm	RNA	Whole Transcriptome	13.2 cm × 13.2 cm	Sequencing	[27]
Visium	100 μm	RNA	Whole Transcriptome	6.5 mm × 6.5 mm	Sequencing	[28]
Xennium	50 nm	RNA	<500	12 mm × 24 mm	Fluorescence Imaging	[29]
ATAC–RNA-seq	20 μm	RNA, Epigenetic	Whole Transcriptome	100 × 100 Barcodes	Fluorescence Imaging	[30]
Epigenomic MERFISH	1 µm	Epigenetic and Protein	<200	Slide Area	Fluorescence Imaging	[31]

**Table 2:** Summary of the applications of spatial omics technologies in AD.

Author/s, Year	Sample	Platform	Findings	References
Muñoz-Castro C et al., 2022	Human temporal association cortex	CyCIF	CyCIF technology enables comprehensive morphological characterization of astrocytes and microglia in the context of their spatial relationships with plaques and tangles in Alzheimer’s Disease brains, revealing three distinct glial phenotypes.	[71]
Muñoz-Castro C et al, 2024	Human temporal lobe	CyCIF	CyCIF technology allows the labeling of up to 16 antigens on the same tissue section, revealing the characterization of astrocytic and microglial responses in Alzheimer’s Disease pathology.	[72]
Lazarian, Artur et al., 2022	APP/PS1 mice coronal-cut brain slices	DESI IMS	DESI Imaging Mass Spectrometry (IMS) can be used to compare lipid species in wild-type (WT) and AD mouse brains across different ages, revealing regional dysregulation, particularly in the hypothalamus.	[73]
Yueguang Lv et al., 2024	APP/PS1 mice brains	DESI IMS	Segmented temperature-controlled DESI (STC-DESI) enables the identification of molecular changes associated with individual Aβ aggregates, revealing potential diagnostic and therapeutic targets such as carnosine and 5-caffeoylquinic acid (5-CQA) in AD pathology.	[74]
Takeyama E et al., 2019	SAMP8 mice Brains	DESI IMS	DESI-IMS can be used to observe the effects of supplementation with DHA-rich green nut oil (GNO) or docosahexaenoic acid (DHA) supplementation on DHA distribution in the brain, providing insights into potential therapeutic strategies for dementia prevention.	[75]
Hashimoto M et al., 2012	Human hippocampal cryosections	LCM-MS	LCM-MS technology can be used to reveal alterations in the proteome of specific neuronal populations, highlighting differences that cannot be detected through bulk analysis methods like Western blotting.	[76]
Bishay J, et al., 2022	Tgf344-AD rat brains	LCM-MS	LCM-MS technology can be used to analyze isolated cortical parenchymal plaques, arteriolar, and venular amyloids, revealing the presence of Aβ and proteins associated with AD in all samples.	[77]
Bishay, Jossana et al., 2020	Tgf344-AD rat brains	LCM-MS	LCM-MS technology can be used to identify amyloid-beta (Aβ) in cortical plaques, CAA, and venular amyloid samples, shedding light on the interplay between these vascular pathologies and AD progression.	[78]
Lars Tjernberg et al., 2011	Human hippocampal cryosections	LCM-MS	LCM-MS technology can be used to analyze alterations in pyramidal neurons from human brains, identify and quantify 150 proteins with altered expression in AD.	[79]
Kaya I et al., 2017	Tgarcswe mice brains	MALDI IMS	The MALDI-IMS paradigm can be used in negative- and positive-ion-mode lipid analysis and subsequent protein ion imaging on the same tissue section, allowing comprehensive, high-resolution molecular analysis of histological features at cellular length scales with high chemical specificity.	[80]
Kakuda N, et al., 2017	Human occipital cortex	MALDI IMS	MALDI-IMS technology enables the visualization of distinct depositions of truncated and/or modified Aβ species, particularly Aβ1–41, in a spacio-temporal specific manner.	[81]
Hong JH et al., 2016	5xFAD mice brains	MALDI IMS	MALDI-IM technology enables the analysis and visualization of lipid profiles in different brain regions affected by AD pathology.	[82]
Kaya I et al., 2018	Tgarcswe mice brains	MALDI IMS	MALDI Imaging Mass Spectrometry (IMS) analyzes and investigates the molecular information associated with individual amyloid aggregates, revealing alterations in lipid species.	[83]
Phongpreecha T et al., 2021	Human brains from individuals with ADNC and LBD; PS/APP and C57Bl6 WT mice brains	MIBI-TOF	MIBI-TOF technology enables the measurement of multiple antibody probes in single-synapse events and provides comprehensive synaptic molecular characterization.	[84]
Vijayaragavan K et al., 2022	Human brains	MIBI-TOF	MIBI-TOF technology enables simultaneous quantitative imaging of multiple proteins in archival human hippocampal tissues, providing insights into neurodegenerative disorders and serving as a methodology for spatial proteomic analysis.	[85]
Moon DW et al., 2020	APP-C105 and 5xFAD Tg mice hippo-campal	Multiplex ion-beam	Multiplex ion-beam technology enables simultaneous imaging of multiple proteins at <300 nm spatial resolution without ion beam damage, revealing insights into protein cluster proximity and its relationship with aging and AD progression.	[86]
Vijayaragavan K et al., 2022	Human Brain	Multiplex ion-beam	multiplex ion-beam technology, enables simultaneous imaging of 36 proteins in human hippocampal tissues across various stages of Alzheimer’s Disease (AD) neuropathologic change, unveiling unique insights into proteopathies and cellular interactions in neurodegenerative disorders.	[85]
Murray HC et al., 2022	Human olfactory bulbs	MP-IHC	Multiplexed fluorescence-based immunohistochemistry (MP-IHC) allows high-content analysis of up to 100 markers on single tissue sections, with application demonstrated in characterizing human olfactory bulb anatomy and identifying differentially expressed biomarkers in Alzheimer’s Disease.	[19]
Ramsden CE et al., 2023	Human ventral entorhinal cortex (erc), prosubiculum (pros)-CA1border region, and temporal neocortex	MP-IHC	MP-IHC allows the characterization of apoer2 expression and the accumulation of pathway components in various regions affected by Alzheimer’s Disease.	[87]
Ramsden CE et al., 2022	Human coronal medial temporal lobe	MP-IHC	MP-IHC technology involves characterizing the expression of apoer2 and apoe in sporadic Alzheimer’s Disease. This study proposes a hypothesis based on lipid peroxidation and the disruption of the apoe/Reelin-apoer2-Dab1 signaling cascade.	[88]
Alyssa Rosenbloom et al., 2023	Adult mouse brain, mouse embryo, and AD-positive human brain	CosMx SMI	The CosMx SMI technology provides a spatial multi-omics platform capable of detecting over 68 proteins at subcellular resolution and capturing the complexity of neuronal and glial cellular activity within their full spatial context.	[89]
Gowoon Son et al., 2024	Human hypothalamus	GeoMX DSP	GeoMX DSP technology reveals that the suprachiasmatic nucleus (SCN) is vulnerable to AD-tau pathology and show immune dysregulation but is protected against ß-amyloid (Aß) accumulation, which may contribute to circadian rhythm disturbances in AD.	[90]
Walker, J.M et al., 2023	The entorhinal cortex, CA1 and CA2 hippocampal subregions	GeoMX DSP	GeoMX DSP technology compares protein expression differences in hippocampal subregions between Alzheimer’s Disease (AD) and primary age-related tauopathy (PART), identifying higher levels of synaptic markers in PART and higher levels of p-tau epitopes in AD.	[91]
Walker, J.M et al., 2020	Human hippocampus	GeoMX DSP	GeoMX DSP technology enables the analysis of protein expression in resilient individuals with Alzheimer’s Disease neuropathologic changes but no cognitive impairment, revealing patterns suggestive of reduced energetic and oxidative stress, increased synaptophysin (SYP) expression and the maintenance of neuronal synapses and connections compared to demented individuals.	[92]
Son, G et al., 2022	Human suprachiasmatic nucleus (SCN), supraoptic nucleus (SON), and periventricular nucleus (PV) in the anterior hypothalamus of brains	GeoMX DSP	GeoMX DSP technology lies in its ability to spatially assess tauopathy and tau-driven protein alterations in the anterior hypothalamic nuclei, especially the suprachiasmatic nucleus (SCN), uncovering elevated p-tau expression in Alzheimer’s Disease (AD) patients compared to controls, which may impact sleep regulation in AD.	[93]
Jose Davila-Velderrain et al., 2021	Human hippocampus and entorhinal cortex	LCM-seq	LCM-seq technology involves conducting a detailed single-cell transcriptomic analysis of the human hippocampus and entorhinal cortex, uncovering cell-type specific transcriptional changes throughout the progression of Alzheimer’s Disease.	[94]
Gabitto MI, et al., 2023	Human superior and middle temporal gyrus (MTG)	MERFISH	MERFISH technology involves utilizing single-cell and spatial genomics tools to investigate the impact of Alzheimer’s Disease progression on cell types in the middle temporal gyrus, revealing temporal changes in neuronal subtypes and glial states, with a subset of donors exhibiting severe cellular and molecular phenotypes correlated with cognitive decline.	[95]
Johnston K, et al., 2023	*Trem2*R47H and 5xFAD mice hippocampus and subiculum	MERFISH	MERFISH technology involves investigating cell-type-specific spatial transcriptomic changes induced by the Trem2R47H mutation in mouse brain sections, revealing consistent upregulation of Bdnf and Ntrk2 across cortical excitatory neuron types and spatially localized reductions in neuronal subpopulations.	[96]
Cable DM, Murray E et al., 2022	Mice hippocampus	Slide-seq	Slide-seq technology involves developing C-SIDE, a framework that models gene expression across cell types, enabling statistical inference for identifying differential expression in various contexts such as pathology, anatomical regions, cell-to-cell interactions, and cellular microenvironment, and it can specifically identify plaque-dependent immune activity in Alzheimer’s Disease and cellular interactions between tumor and immune cells.	[97]
Guang-Wei Zhang et al., 2023	5xFAD mouse Brains	Sstereo-seq	Stereo-seq technology enables us to spatially profile whole-genome transcriptomics in the 5xfad mouse model, establishing a methodology for analyzing specific neuronal transcriptomic changes spatially correlated with amyloid pathology at single-cell resolution.	[98]
Shuo Chen, Yuzhou Chang et al., 2022	Human brains	Visium	Visium identifies unique marker genes for cortical layers and white matter, layer-specific differentially expressed genes (degs) in AD, and illustrates significant differences in specific cell types among cortical layers and white matter regions.	[28]
Sang Ho Kwon et al., 2023	Human brains	Visium	Visium technology enables us to assess spatial gene expression changes in AD brains relative to Aβ and hyperphosphorylated tau (ptau) pathology, revealing transcriptomic signatures associated with Aβ proximity in late-stage AD.	[99]
Hongyoon Choi et al., 2023	5xFAD mice brains	Visium	Visium technology reveals regional changes at the molecular level, including early alterations in the white matter (WM) involving glial cell activation before the accumulation of amyloid plaques in the gray matter (GM), and identifying distinct spatial patterns of disease-associated microglia (dams).	[100]
Alon Millet, Jose Henrique Ledo et al., 2024	5xFAD mice brain cortex and hippocampus; human brain of age-matched Alzheimer’s Disease APOE3/APOE3 carriers and APOE4/APOE4 carriers	Xenium	Xenium technology enables us to identify a reactive microglial population termed terminally inflammatory microglia (tims) characterized by inflammatory signals and cell-intrinsic stress markers, whose frequency increased with age and APOE4 burden, and was detectable in human AD patients’ brains.	[101]

**Table 3:** Comparative analysis of spatial omics technologies.

Items	Proteomics in Bodily Fluids (CSF, Blood)	Spatial Proteomics	Bulk Transcriptome	Single-Cell Transcriptome	Spatial Transcriptome
Analytic object	CSF, blood	Tissue	Tissue	Cell	Tissue
Region	Not applicable	Identified directly	Not applicable	Presumed by the algorithm	Identified directly
Advantage	Wide applicabilityLow sample size	Spatial resolution Direct visualization	The price is low	Cell resolution	Spatial resolution
Limitation	Lack of spatial information	Sample volumesLong duration and limited throughput	Precision is low Lack of spatial information Resolution is low	Lack of spatial information	The resolution and hroughput are lower than single-cell transcriptome in most cases
